# Novel Surgical Technique of Liver Parenchyma Transection During Robotic‐Assisted Liver Resection: “Trac & Pac”

**DOI:** 10.1002/jhbp.70006

**Published:** 2025-08-22

**Authors:** Yuta Abe, Yutaka Nakano, Yosuke Uematsu, Minoru Kitago, Yasushi Hasegawa, Shutaro Hori, Masayuki Tanaka, Taizo Hibi, Yuko Kitagawa

**Affiliations:** ^1^ Department of Surgery Keio University School of Medicine Tokyo Japan; ^2^ Department of Pediatric Surgery and Transplantation Kumamoto University Graduate School of Medical Sciences Kumamoto Japan

**Keywords:** Cavitron Ultrasonic Surgical Aspirator, liver parenchyma dissection, robotic‐assisted liver resection, Trac & Pac

## Abstract

**Background/Purpose:**

A standardized liver parenchymal transection method in robotic‐assisted liver resection has not yet been established, and the techniques used vary among institutions. We developed a novel transection technique for robotic‐assisted liver resection termed “Trac & Pac,” which uses maryland bipolar forceps with a gentle stroking motion and strategic traction to progressively expose and dissect the liver parenchyma.

**Methods:**

We described the technical details of “Trac & Pac” and Evaluated its short‐term outcomes (2022–2025), comparing them with those of conventional laparoscopic liver resection using the Cavitron Ultrasonic Surgical Aspirator (CUSA) (2018–2022).

**Results:**

The robotic‐assisted liver resection group included 26 patients who underwent robotic left or right hepatectomy with the “Trac & Pac” technique, whereas the laparoscopic liver resection group comprised 34 patients who underwent laparoscopic left or right hepatectomy using the CUSA. The robotic group had a longer time from pneumoperitoneum to the start of intra‐abdominal manipulation than the laparoscopic group (*p* < 0.001). Both groups had similar parenchymal transection times, blood loss, and postoperative complications.

**Conclusions:**

“Trac & Pac” is a safe and feasible robotic‐assisted liver parenchymal transection technique that may provide a new solution for improving precision and exposure in minimally invasive liver surgery.

## Graphical abstract

Abe and colleagues introduce “Trac & Pac,” a novel robotic‐assisted liver transection technique. In 26 robotic cases, outcomes were comparable to those of 34 conventional laparoscopic cases using CUSA, with similar transection time, blood loss, and complications, demonstrating the technique's safety, feasibility, precision, and potential to standardize robotic liver surgery.

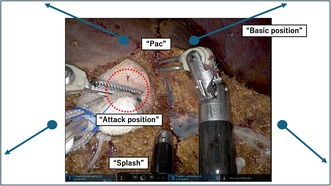

## 1 Introduction

Robotic‐assisted liver resection (RLR) has recently been increasingly adopted, particularly at high‐volume centers, due to advancements in robotic technology and the accumulation of surgical expertise. Regarding its short‐term outcomes, some studies have shown that RLR is associated with lower intraoperative blood loss and a shorter postoperative hospital stay than laparoscopic liver resection (LLR) [1, 2, 3, 4] owing to its advantages, including articulated instruments and stable imaging capabilities.

However, one of the major challenges of RLR is the lack of a standardized method for liver parenchymal transection. A survey of devices used for RLR among 800 members of the European‐African Hepato‐Pancreato‐Biliary Association conducted between 2019 and 2020 revealed that robotic bipolar devices, assistant‐operated Cavitron Ultrasonic Surgical Aspirator (CUSA), robotic vessel sealers, and robotic ultracision were used in 30%, 25%, 20%, and 18% of cases, respectively, indicating a significant variation in transection techniques across institutions [5]. In the survey, 70% of RLR surgeons responded that they would prefer a CUSA that could be operated on directly by the console surgeon [5]. However, the inability to use CUSA in the current robotic setup highlights parenchymal transection as a critical and unresolved issue in RLR.

Our institution has adopted a novel liver parenchymal transection technique, “Trac & Pac,” which employs Maryland bipolar forceps and could serve as an alternative to CUSA for robotic hepatectomy. In this study, we initially provided a detailed description of the “Trac & Pac” technique and subsequently compared the short‐term surgical outcomes of RLR using this technique with those of LLR using CUSA for both left and right hepatectomies, aiming to evaluate the safety and feasibility of this novel approach.

## 2 Methods

### 2.1 Patients

The data of patients who underwent RLR between August 2022 and March 2025 were retrospectively reviewed. Among these patients, those who underwent robotic left or right hepatectomy using the “Trac & Pac” technique were selected to compare the short‐term operative outcomes with those of patients who underwent laparoscopic left or right hepatectomy with the CUSA between January 2018 and August 2022.

This study was conducted in accordance with the principles of the 1975 Declaration of Helsinki and was approved by the Ethics Committee of Keio University School of Medicine (approval number: 20120443). The requirement for informed consent was waived owing to the retrospective nature of this study.

### 2.2 Surgical Technique of “Trac & Pac”

#### 2.2.1 Concept of “Trac & Pac”

The name “Trac & Pac” originates from two key ideas: “Trac,” which highlights the crucial role of traction during liver resection and “Pac,” referring to the liver transection motion, which resembles the opening and closing action of the Japanese character “Pac‐Man.” Together, the name merges these two concepts. The “Trac & Pac” technique is conceptually similar to CUSA‐based transection, with a focus on the meticulous excavation of vessels [6]. Similar to archaeological excavations where buried remains are unearthed and exposed in an intact form, excavation is the important concept of liver parenchymal transection with CUSA. Ultrasonic vibration gently fragments the liver parenchyma at the tip in the case of CUSA. CUSA provides some hemostatic capacity through ultrasonic vibration, which can be augmented by soft coagulation. It also provides suction for excess fluid and blood, allowing the surgeon to clearly visualize, isolate, and follow the vessels during transection [6]. The “Trac & Pac” technique follows a principle similar to that of CUSA for liver parenchymal transection. Technical details of this method are described in the following sections.

#### 2.2.2 Contents of “Trac & Pac”

The contents of the “Trac & Pac” technique are described below.

##### 2.2.2.1 “Pac”: Moving and Handling Maryland Bipolar Forceps

“Gentle stroking,” rather than clamping, is the fundamental principle of using the Maryland bipolar forceps in the “Trac & Pac” technique. Instead of grasping the tissue between the blades, each blade independently strokes the liver parenchyma and gradually separates it into small increments. This mechanical action closely mimics the effect of ultrasonic vibrations in CUSA. During the opening and closing motions, the blades apply a gentle shearing or rubbing force that mechanically dissects the tissue. Bipolar energy is applied between the blades to provide controlled coagulation and dissection, similar to the soft coagulation function of CUSA. Only the distal one‐fourth of the blade tips are used for dissection, enabling precise and delicate manipulation. Although the Clamp crushing maneuvers are applied selectively, when necessary, the use of coagulation during these steps should be avoided to prevent unnecessary tissue damage. “Bipolar Soft” is the preferred energy mode, as it avoids settings like “macro mode” that may produce sparks or uncontrolled cutting [7, 8]. In this technique, tissue transection is primarily achieved through the physical action of the blades rather than through energy‐based cutting. The forceps gently separate the coagulated parenchyma, thereby maintaining hemostasis.

**FIGURE 1:**
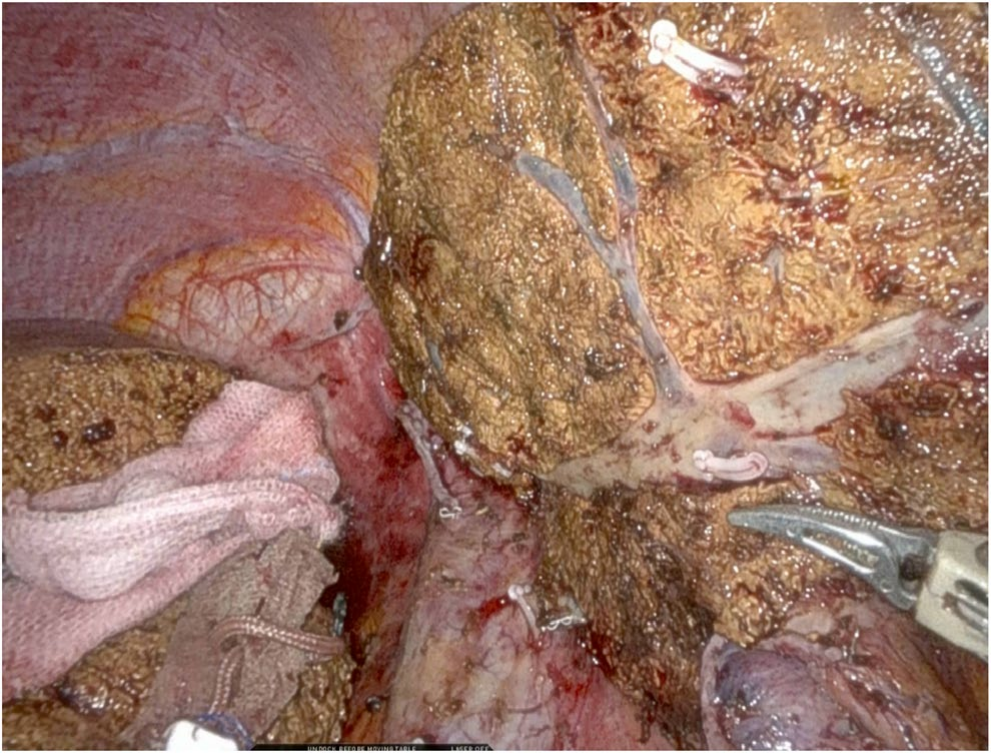
Intraoperative view of the exposed middle hepatic vein. The “Trac & Pac” technique enables vascular exposure and hepatic vein tracking at a level comparable to other methods.

There are no substantial differences in the handling of devices or forceps between parenchymal dissection and vascular structure exposure. By applying traction and gradually disrupting the parenchyma using the Pac motion, vessels can be visualized from within the liver parenchyma and isolated by clearing the surrounding tissue (Figure 1). Similarly, during exposure and tracing of hepatic veins, the Pac motion is primarily used to stroke gently along the vein surface. However, the Peel, which is the basic way of dissection, and Sweep, which is the motion of tracing the cut surface gently, are also effective in this context. Pac, Sweep, and Peel share the common characteristic of using the tip of the blade to stroke the tissue gently (Video S1).

There is no intention to emphasize the “speed” of the motion. Regarding the precision of the robotic forceps, the tip control offered by robotic instruments surpasses that of laparoscopic instruments. Regardless of how the absolute precision of the robot is evaluated, it is more stable and precise than laparoscopy, which may offer advantages in parenchymal transection. Notably, the Pac motion cannot be reproduced with laparoscopic instruments as their unstable tips make it difficult to repeatedly and accurately stroke the same location.

##### 2.2.2.2 “Traction”: Creating the Surgical Field During RLR

**FIGURE 2:**
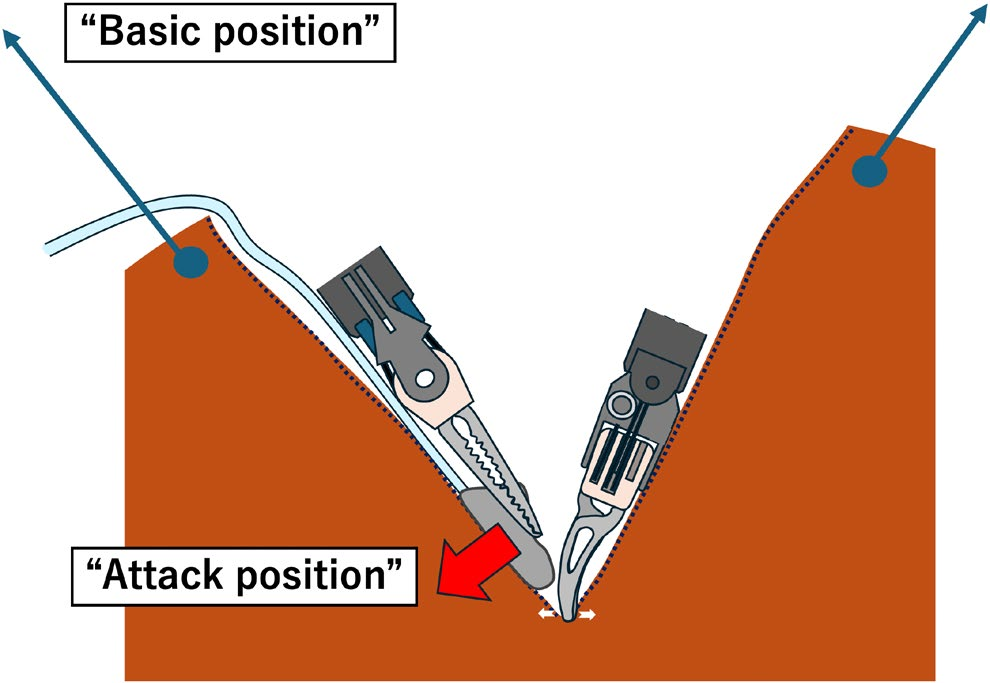
Schema of the “Basic position” and “Attack position.” “Basic position” refers to the initial entire traction applied to achieve a stable and broad exposure before initiating liver parenchyma transection using a traction arm or stay sutures. “Attack position” involves fine countertraction using A‐Suc, a continuous suction sponge held in the left hand, to gently manipulate and apply tension to a specific portion of the liver parenchyma targeted for transection.

Adequate traction and proper exposure to the surgical field are the most critical components of the “Trac & Pac” technique. Clear visualization of the transection plane is essential for achieving precise and safe liver parenchymal dissection. The procedure begins by applying global tension to the liver using a traction arm or stay sutures to provide stable and broad exposure, referred to as the “Basic Position.” Next, fine countertraction is applied using a protective material, such as gauze or a sponge, with forceps held in the contralateral arm to gently manipulate and apply tension to a specific portion of the liver parenchyma targeted for dissection, known as the “Attack Position.” In this technique, the liver parenchyma dissection is not primarily driven by the right‐hand instrument but rather facilitated by the countertraction from the contralateral arm (Figure 2). This controlled exposure, combined with the gentle stroking motion of the Maryland bipolar forceps, which referred to as the “Pac,” enables the liver parenchyma to be dissected smoothly and in a controlled manner. The robotic system's precision and stability, which minimizes instrument tip tremors, further enhance the surgeon's ability to identify and preserve small vascular structures, thereby enabling refined and meticulous liver parenchymal transection. Video S2 demonstrates the “Pac” and “Trac.”

##### 2.2.2.3 “Splash”: Using Saline to Clean the Surgical Field and Stop Bleeding at a Specific Point

**FIGURE 3:**
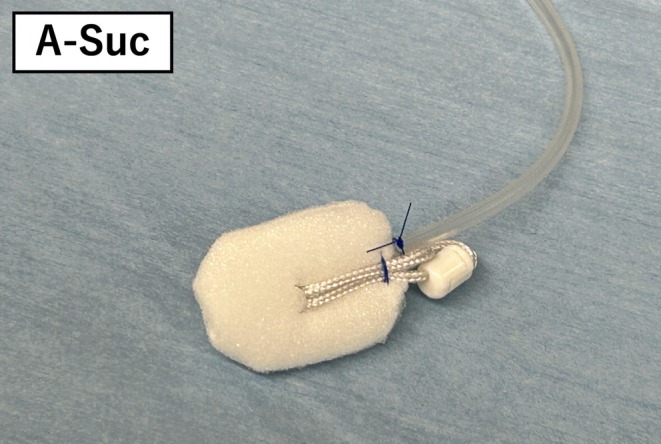
A‐Suc is an original continuous suction sponge, which is used to make the “attack position” and remove the excess fluid.

An essential responsibility of the assistant during the “Trac & Pac” technique is managing the irrigation, commonly known as “Splash.” This maneuver fulfills two critical functions. First, it identifies the specific bleeding point and prevents carbonization and tissue sticking on the Maryland bipolar forceps. Bipolar energy is fundamentally dependent on the presence of saline. The tips of the forceps tend to overheat and become carbonized in the absence of adequate irrigation. This carbonization causes tissue to adhere to the blades, which, in turn, causes the tissue to tear rather than separate cleanly, potentially leading to unexpected bleeding. Field visualization also becomes difficult when blood mixes with coagulated tissue on the transection surface. If the surgeon continues dissection under these conditions, the coagulated blood can form dark, charred clots that obscure the anatomy and interfere with precise manipulation. Therefore, selective and timely irrigation with saline is essential to clearly delineate bleeding points and maintain a clean operative field. However, excessive irrigation is problematic. When excess saline accumulates, it reduces the electrical conductivity, thereby compromising the efficacy of the bipolar device (Video S3). Maintaining adequate fluid control in the surgical field is essentail, and the “Trac & Pac” technique similarly requires an environment that allows for simultaneous irrigation and suction, as achieved with the CUSA. To facilitate this, irrigation is performed by the assistant, whereas self‐controlled suction is achieved using the A‐Suc. A‐Suc is an original continuous suction sponge used to make the “attack position” and remove the excess fluid (Figure 3). Although the assistant can perform suction, the range of motion is limited by the robotic arms, which makes it difficult to suction as freely as in LLR. Consequently, the assistant primarily focuses on administering normal saline, whereas the surgeon controls the fluid in the surgical field using the A‐Suc. This approach is functionally similar to the suction mechanism of CUSA, which maintains a delicate balance between adequate irrigation and effective coagulation.

#### 2.2.3 Differences Between the “Trac & Pac” and “Clamp Crushing” Techniques

**FIGURE 4:**
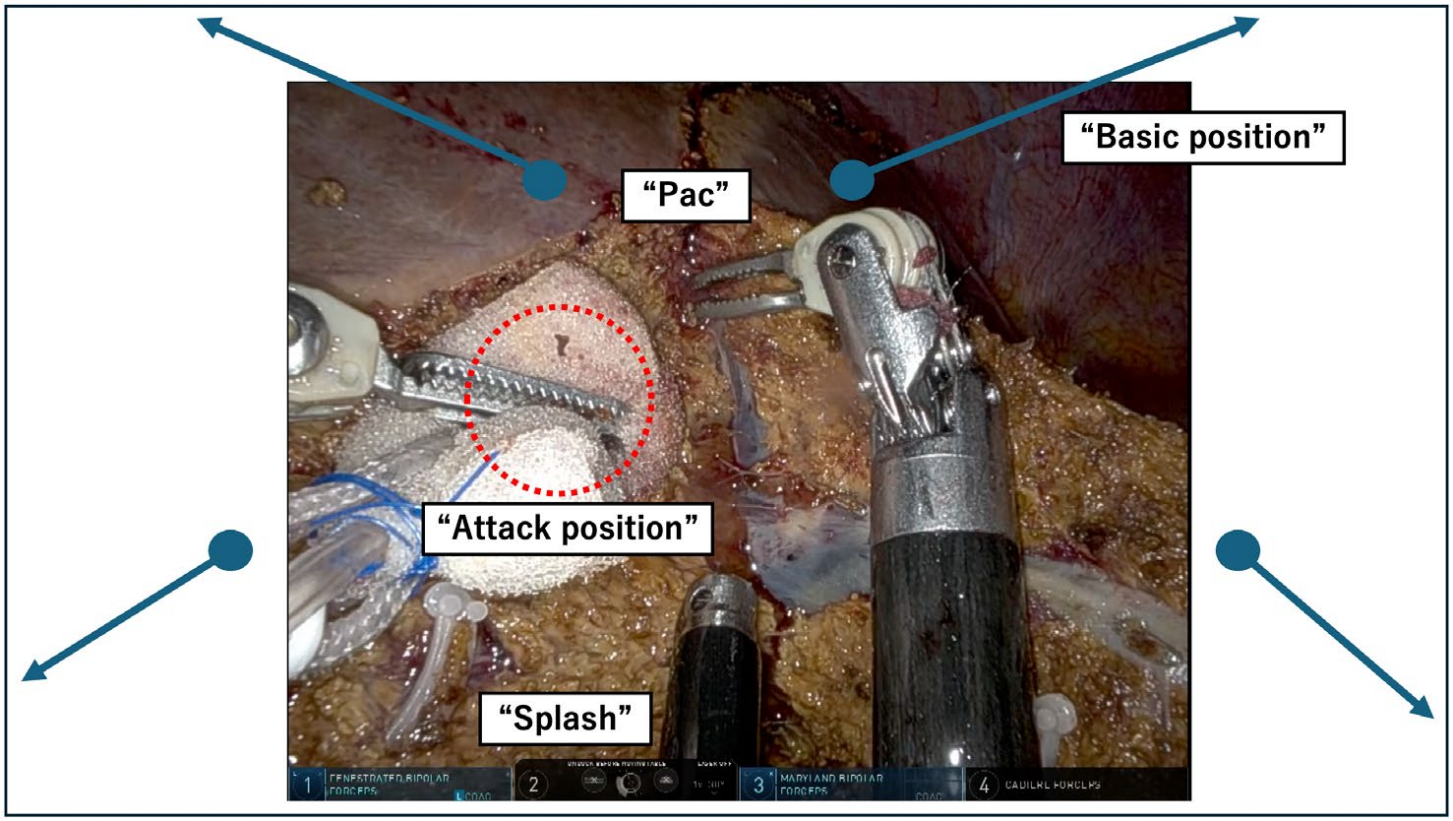
Intraoperative view of the “Trac & Pac” technique: “Pac,” “Traction,” and “Splash.” The “Trac & Pac” technique consists of “Pac,” “Traction (Basic and Attack positions),” and “Splash.” The synergy of these three elements is essential for the execution of the “Trac & Pac” technique.

**FIGURE 5:**
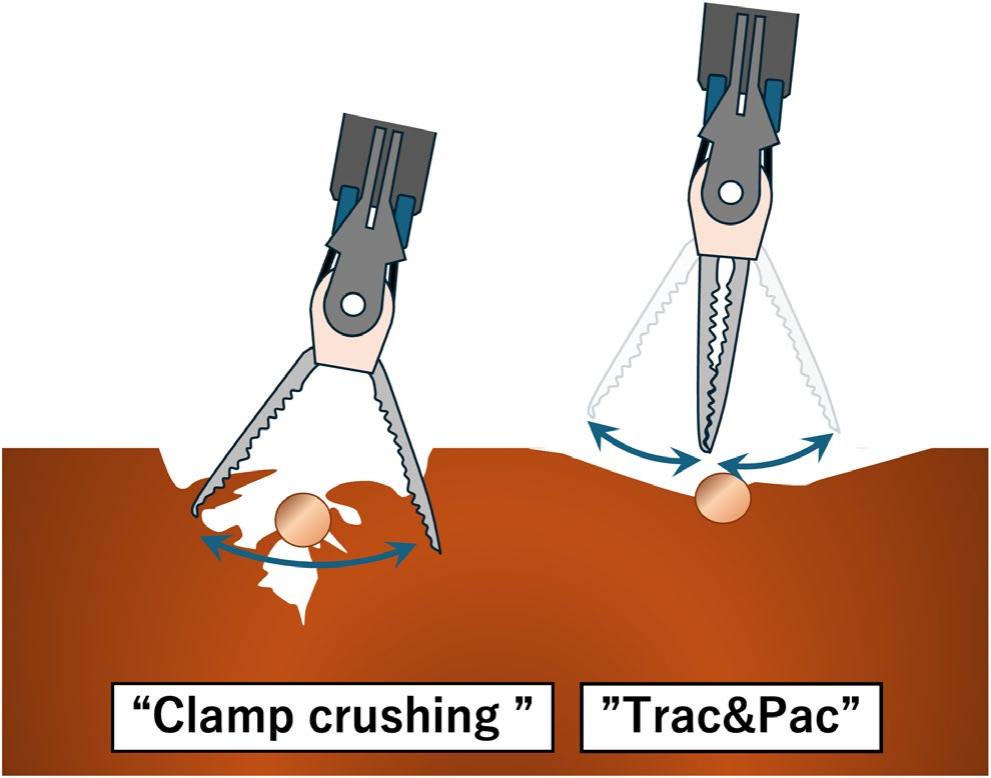
Schema of the “Clamp crushing” and “Trac & Pac” techniques. The “Clamp crushing” technique is performed by grasping and crushing large segments, after which vascular structures are subsequently identified and managed within the crushed parenchyma. In contrast, the “Trac & Pac” technique does not involve large‐scale crushing of the parenchyma. It is performed by gently stroking from the anterior side, breaking down tissue incrementally as the operative field advances, ensuring that vascular structures are exposed ahead of the dissection plane.

Although the “Trac & Pac” technique (Figure 4) may appear similar to the conventional “Clamp crushing” method at first glance, the underlying principles and mechanisms are fundamentally different. The liver parenchyma is grasped and crushed in large segments in the “Clamp crushing” technique. Vascular structures within the crushed parenchyma are subsequently identified and managed. This technique typically involves a single forceful clamping motion without the simultaneous application of bipolar energy. In contrast, the “Trac & Pac” technique does not involve large‐scale crushing of the liver parenchyma. As previously described, Maryland bipolar forceps are used to gently stroke and gradually break down the tissues. With this approach, the dissection progresses from the anterior side by incrementally breaking down tissue as the operative field advances, ensuring that vascular structures are exposed ahead of the dissection plane rather than being trapped in a crushed mass as observed in the “Clamp crushing” technique (Figure 5).

### 2.3 Statistical Analyses

Surgical outcomes were analyzed using operative records. Each operative video was assessed by two expert surgeons in the hepatobiliary‐pancreatic field to collect data, including the Pringle maneuver and liver mobilization times. Categorical and continuous variables were compared using the chi‐square or Fisher's exact test and the Mann–Whitney *U*‐test, respectively. All statistical analyses were conducted using the IBM SPSS Statistics for Macintosh, version 29.0 (IBM Corp., Armonk, NY, USA). Statistical significance was set at *p* < 0.05.

## 3 Results

In total, 24 robotic left or right hepatectomies using the “Trac & Pac” technique (12 left and 14 right) (RLR group) and 34 laparoscopic left or right hepatectomies using CUSA (24 left and 10 right) (LLR group) were included. No significant differences in background factors were observed between the groups, except for body mass index in left hepatectomy (Tables 1 and 2). Liver mobilization time was significantly shorter in the RLR group than in the LRL group for both left and right hepatectomies. Although the time from pneumoperitoneum to the start of intra‐abdominal manipulation was significantly longer in the RLR group than in the LLR group (*p* < 0.001), the parenchymal transection time, blood loss, postoperative hospital stay, and postoperative complications were similar between the groups for both left and right hepatectomies (Tables 3 and 4).

**TABLE 1:** Characteristics of patients who underwent left hepatectomy.

Variables	Total	RLR	LLR	*p*
	*n* = 36	*n* = 12	*n* = 24	
Patients' characteristics				
Age (years)	68 (37–86)	61 (37–85)	69 (37–86)	0.282
Sex male	20 (55.5)	6 (50.0)	14 (58.3)	0.635
Body mass index (kg/m^2^)	22.8 (15.8–32.0)	25.5 (15.8–32.0)	21.6 (16.5–29.8)	0.032
Diagnosis				
Hepatocellular carcinoma	16 (44.5)	4 (33.3)	12 (50.0)	0.520
Liver metastasis	7 (19.4)	2 (16.7)	5 (20.8)	
Intrahepatic cholangiocarcinoma	7 (19.4)	4 (33.3)	3 (12.5)	
Others	6 (16.7)	2 (16.7)	4 (16.7)	
ASA‐PS ≥ 3	6 (16.7)	1 (8.3)	5 (20.8)	0.319
Child‐Pugh score				
5	33 (91.7)	12 (100.0)	21 (87.5)	0.109
6	3 (8.3)	0 (0.0)	3 (11.5)	
ICG15R (%)	8.0 (1.0–22.7)	10.1 (1.0–20.6)	7.8 (2.3–22.7)	0.139
Repeat hepatectomy	1 (2.7)	1 (8.3)	0 (0.0)	0.133

*Note:* Continuous variables are presented as medians (range), whereas categorical variables are presented as *n* (%) unless otherwise noted.

Abbreviations: ASA‐PS, American Society of Anesthesiologists Physical Status classification system; ICG‐R15; indocyanine green retention rate at 15 min; LLR, laparoscopic liver resection; RLR, robotic‐assisted liver resection.

**TABLE 2:** Characteristics of patients who underwent right hepatectomy.

Variables	Total	RLR	LLR	*p*
	*n* = 24	*n* = 14	*n* = 10	
Patients' characteristics				
Age (years)	65 (24–81)	66 (24–81)	64 (58–81)	0.659
Sex male	13 (54.1)	6 (42.9)	7 (70.0)	0.184
Body mass index (kg/m^2^)	21.3 (15.3–33.5)	21.3 (15.3–33.5)	20.8 (16.6–26.2)	0.861
Diagnosis				
Hepatocellular carcinoma	10 (41.6)	5 (35.7)	5 (50.0)	0.308
Liver metastasis	9 (37.5)	5 (35.7)	4 (40.0)	
Intrahepatic cholangiocarcinoma	2 (12.5)	1 (7.1)	1 (10.0)	
Others	3 (12.5)	3 (21.4)	0 (0.0)	
ASA‐PS ≥ 3	2 (8.3)	0 (0.0)	2 (20.0)	0.052
Child‐Pugh score				
5	23 (95.8)	14 (100.0)	9 (90.0)	0.178
6	1 (4.2)	0 (0.0)	1 (10.0)	
ICG15R (%)	9.3 (1.6–18.0)	10.1 (3.4–18.0)	7.5 (1.6–14.7)	0.266
Repeat hepatectomy	1 (4.2)	0 (0.0)	1 (10.0)	0.178
Portal Vein Embolization	10 (41.7)	4 (28.6)	6 (60.0)	0.122
Preoperative simulation (mL)				
Standard liver volume	1130 (932–1515)	1129 (932–1515)	1135 (952–1260)	0.907
Total liver volume	1102 (720–2210)	1023 (720–2415)	1169 (942–1651)	0.197
Estimated resected liver volume	580 (300–1369)	542 (300–1747)	672 (393–972)	0.639

*Note:* Continuous variables are presented as medians (range), whereas categorical variables are presented as *n* (%) unless otherwise noted.

Abbreviations: ASA‐PS, American Society of Anesthesiologists Physical Status classification system; ICG‐R15; indocyanine green retention rate at 15 min; LLR, laparoscopic liver resection; RLR, robotic‐assisted liver resection.

**TABLE 3:** Intraoperative and postoperative outcomes in the left hepatectomy group.

Variables	Total	RLR	LLR	*p*
	*n* = 36	*n* = 12	*n* = 24	
Surgical outcome				
Surgeon; expert	21 (58.3)	8 (66.7)	13 (54.2)	0.473
Operation time (min)	393 (203–794)	385 (274–782)	397 (203–794)	0.551
Blood loss (mL)	8 (5–600)	5 (5–400)	45 (5–600)	0.073
Total Pringle maneuver time (min)	96 (47–184)	88 (47–184)	110 (47–176)	0.168
Details of the surgery				
Inflow control				
Glissonean approach	22 (61.1)	5 (41.7)	17 (70.8)	0.092
Individual hilar dissection	14 (38.9)	7 (58.3)	7 (29.2)	
Resection with the middle hepatic vein	5 (13.8)	3 (25.0)	2 (8.3)	0.186
Exposure of the middle hepatic vein	27 (75.0)	10 (83.3)	17 (70.8)	0.402
Combined resection of the Spiegel lobe	10 (27.7)	6 (50.0)	4 (16.7)	0.038
Lymph node dissection or sampling	9 (25.0)	4 (33.3)	5 (20.8)	0.420
Time for each surgical process (min)				
Pneumoperitoneum to start of intra‐abdominal manipulation	7 (3–32)	24 (20–32)	4 (3–13)	< 0.001
Liver mobilization	24 (11–80)	25 (11–55)	23 (14–80)	0.724
Inflow control and lymph node dissection^a^	38 (12–173)	49 (17–173)	34 (12–141)	0.107
Outflow control^b^	16 (3–44)	15 (9–36)	16 (3–44)	0.602
Liver parenchymal transection	81 (42–173)	79 (50–173)	84 (42–173)	0.987
Postoperative outcome				
Clavien‐Dindo classification ≥ IIIa^c^	2 (5.5)	1 (8.3)	1 (4.1)	0.617
Postoperative bile leakage ≥ Grade B^d^	0 (0.0)	0 (0.0)	0 (0.0)	—
Readmission ≤ 30 days	1 (2.7)	1 (8.3)	0 (0.0)	0.133
90‐day or inhospital mortality	0 (0.0)	0 (0.0)	0 (0.0)	—
Postoperative hospital stays (days)	10 (7–63)	11 (8–19)	9 (7–63)	0.285
Pathological findings				
Tumor size (mm)	35 (20–115)	31 (22–41)	36 (20–115)	0.094
Number of tumors	1 (1–9)	1 (1–1)	1 (1–9)	0.131
R0 resection margin	35 (97.2)	11 (91.7)	24 (100.0)	0.133
Fibrosis ≥ 3^e^	2 (5.6)	2 (16.7)	0 (0.0)	0.031

*Note:* Continuous variables are presented as medians (range), whereas categorical variables are presented as *n* (%) unless otherwise noted.

Abbreviations: LLR, laparoscopic liver resection; RLR, robotic‐assisted liver resection.

^a^ Inflow control was performed using the Glissonean approach or individual hilar dissection. The time required for these procedures was also included in cases where lymph node dissection or sampling was performed.

^b^ Outflow control includes the time required for dissection, taping, and transection at the root of the hepatic vein.

^c^ The grading of postoperative bile leakage was based on the criteria defined by the International Study Group of Liver Surgery.

^d^ Postoperative complications of Clavien–Dindo grade ≥ IIIa included port‐site bleeding and intra‐abdominal abscess in the RLR and LLR groups, respectively.

^e^ METAVIR scoring system.

**TABLE 4:** Intraoperative and postoperative outcomes in the right hepatectomy group.

Variables	Total	RLR	LLR	*p*
	*n* = 24	*n* = 14	*n* = 10	
Surgical outcome				
Surgeon; expert	14 (58.3)	10 (71.4)	4 (40.0)	0.122
Operation time (min)	526 (389–794)	551 (405–794)	490 (389–644)	0.292
Blood loss (mL)	5 (5–200)	5 (5–200)	95 (5–200)	0.124
Total Pringle maneuver time (min)	98 (47–166)	102 (65–166)	99 (47–136)	0.334
Details of the surgery				
Anterior approach	21 (87.5)	14 (100.0)	7 (70.0)	0.015
Resection with the middle hepatic vein	3 (12.5)	2 (14.3)	1 (10.0)	0.751
Exposure of the middle hepatic vein	18 (75.0)	11 (78.6)	7 (70.0)	0.634
Lymph node dissection or sampling	2 (8.4)	1 (7.1)	1 (10.0)	0.804
Time for each surgical process (min)				
Pneumoperitoneum to the start of intra‐abdominal manipulation	20 (3–27)	25 (20–27)	4 (3–6)	< 0.001
Liver mobilization	54 (22–148)	40 (22–148)	68 (40–100)	0.008
Inflow control and lymph node dissection^a^	94 (37–188)	81 (37–188)	105 (70–136)	0.159
Outflow control^b^	19 (11–107)	20 (13–107)	17 (11–34)	0.277
Liver parenchymal transection	94 (52–155)	94 (60–155)	94 (52–140)	0.769
Postoperative outcome				
Clavien‐Dindo classification ≥ IIIa^c^	4 (16.7)	3 (21.4)	1 (10.0)	0.447
Postoperative bile leakage ≥ Grade B^d^	1 (4.2)	1 (7.1)	0 (0.0)	0.292
Readmission ≤ 30 days	1 (4.2)	1 (7.1)	0 (0.0)	0.292
90‐day or inhospital mortality	0 (0.0)	0 (0.0)	0 (0.0)	—
Postoperative hospital stays (days)	11 (8–34)	11 (8–34)	12 (9–31)	0.791
Pathological findings				
Tumor size (mm)	50 (11–170)	52 (11–170)	47 (16–95)	0.660
Number of tumors	1 (1–30)	1 (1–4)	1 (1–30)	0.487
R0 resection margin	23 (95.8)	14 (100.0)	9 (90.0)	0.178
Fibrosis ≥ 3^e^	4 (16.7)	2 (14.3)	2 (20.0)	0.712

*Note:* Continuous variables are presented as medians (range), whereas categorical variables are presented as *n* (%) unless otherwise noted.

Abbreviations: LLR, laparoscopic liver resection; RLR, robotic‐assisted liver resection.

^a^ Inflow control was achieved using individual hilar dissections in all cases. The recorded times comprise the isolation and transection of the right hepatic artery, right portal vein, and hilar plate, including the right hepatic duct. Additionally, the time required for these procedures was also included in cases where lymph node dissection or sampling was performed. Cholecystectomy‐related procedures were excluded.

^b^ Outflow control includes the time required for dissection, taping, and transection at the root of the hepatic vein.

^c^ The grading of postoperative bile leakage was based on the criteria defined by the International Study Group of Liver Surgery.

^d^ Postoperative complications of Clavien–Dindo grade ≥ IIIa included bile leakage, port‐site hernia, intra‐abdominal abscess in the RLR group, and ascitic fluid accumulation in the LLR group.

^e^ METAVIR scoring system.

## 4 Discussion

In this study, we introduced and evaluated a novel liver parenchymal transection technique for RLR, known as the “Trac & Pac” technique, which leverages the unique capabilities of a robotic system to enable the precise and controlled dissection of the liver parenchyma. Compared to conventional techniques such as the “Clamp crushing,” the “Trac & Pac” method employs a combination of strategic countertraction and gentle stroking movements with Maryland bipolar forceps to progressively break down tissue from the anterior side, exposing vascular structures as the dissection advances. The technique also incorporates controlled irrigation and suction to optimize visibility and hemostasis throughout the procedure. Our findings demonstrate that this method is both technically feasible and results in surgical outcomes comparable to those of LLR using CUSA. The “Trac & Pac” technique represents a safe, reproducible, and refined approach for robotic‐assisted liver parenchymal transection, which may help standardize this currently inconsistent step in RLR.

RLR has gained global acceptance across various surgical procedures, with numerous studies reporting favorable outcomes. In an international multicenter study using propensity score‐matching analysis, Krenzien et al. found that robotic‐limited liver resections of the posterosuperior segments had better outcomes than laparoscopic approaches [9]. Liu et al. validated the safety and efficacy of robotic major hepatectomies through a large‐scale analysis of 4822 cases [10]. Furthermore, Hu et al. confirmed the advantages of robotic‐assisted surgery in 10 517 patients by focusing on minor liver resection of the anterolateral segments [11]. Although establishing a standardized liver parenchymal transection technique remains a critical challenge for the further expansion of RLR [5], we believe that the “Trac & Pac” technique could serve as a valuable adjunct to facilitate the global adoption of RLR globally.

With advancements in robotic surgical technology, its application has expanded to highly complex hepatobiliary procedures, including robotic donor hepatectomy [12], recipient liver transplantation [13], and perihilar cholangiocarcinoma [14]. Raptis et al. recently demonstrated that robotic‐assisted living donor right hepatectomy, using real time indocyanine green fluorescence cholangiography, was associated with fewer complications for both the donor and recipient than the standard open approach, highlighting the growing feasibility of robotic platforms for robotic‐assisted living donor hepatectomies [12]. A systematic review by Aoyagi et al. reported that robotic‐assisted surgery for perihilar cholangiocarcinoma shows promise in selected cases, with favorable short‐term outcomes and acceptable morbidity [14]. In this context, the “Trac & Pac” technique may represent a meaningful advancement in addressing one of the key technical barriers in RLR—the lack of a standardized and effective parenchymal transection method. Furthermore, this technique may facilitate safer and broader applications of robotic‐assisted surgery in challenging hepatobiliary cases. The continued development and standardization of such techniques will be critical for expanding the role of robotics in highly complex liver procedures. Moreover, the Trac & Pac technique allows for liver transection within a comparable timeframe to LLR by CUSA, suggesting its potential suitability for complex procedures.

Given that the CUSA, the most commonly used device for laparoscopic liver parenchymal transection, cannot be used in robotic surgery, the ability of the Trac & Pac method to achieve comparable vascular exposure and hepatic vein tracking is highly advantageous. This technique enables many surgeons to perform robotic liver parenchymal transection with a quality equivalent to that of laparoscopic procedures. Furthermore, its comparable transection time to that of CUSA is also a critical factor for the widespread adoption of this technique. Harmonic devices are commonly used in robotic liver resections for their efficiency in tissue cutting and hemostasis. However, because they lack an articulating system, they do not fully leverage the advantages of robotic surgery similar to the limitations observed with CUSA. In contrast, the “Trac & Pac” technique fully utilizes the robotic platform's strengths by enabling precise parenchymal transection using the articulating bipolar forceps under traction.

This study had some limitations. First, this was a retrospective analysis conducted at a single institution, which may introduce a selection bias and limit the generalizability of the findings. Second, although we compared the “Trac & Pac” technique with the well‐established LLR using CUSA, the choice of surgical approach was not randomized, and inherent differences in case selection or surgeon preference may have existed. Third, the number of patients in the group using the “Trac & Pac” technique was relatively small, reflecting the early phase of adoption. Larger multicenter studies are needed to validate the reproducibility and long‐term outcomes of this technique. Fourth, surgical experience and the learning curve may have improved over time, potentially influencing outcomes in the RLR group. To minimize this bias, only cases performed after the adoption of a standardized surgical technique were included, and all participating surgeons had already acquired sufficient experience in both laparoscopic and robotic liver surgery. Despite these efforts, the retrospective nature of the study and the temporal discrepancy between groups remain limitations. Finally, the learning curve for the “Trac & Pac” technique remains to be evaluated as it involves precise instrument handling and coordination between the surgeon and assistant.

In conclusion, a novel liver parenchymal transection technique for RLR, known as “Trac & Pac,” is a safe and feasible procedure compared to LLR using CUSA. Consequently, robotic‐assisted surgery may serve as a new solution for complex LLRs, potentially expanding the range of indications and improving surgical feasibility.

## Conflicts of Interest

The authors declare no conflicts of interest.

## Supporting information


**Video S1:** How to expose and track the hepatic veins.

**Video S2:** “Pac” (moving and handling Maryland bipolar forceps), and “Trac” (creating the surgical field).

**Video S3:** “Splash” (using saline to clean the surgical field and stop bleeding at a specific point).

## Data Availability

The data that support the findings of this study are available from the corresponding author upon reasonable request.
